# Examining the Effect of Reverse Worded Items on the Factor Structure of the Need for Cognition Scale

**DOI:** 10.1371/journal.pone.0157795

**Published:** 2016-06-15

**Authors:** Xijuan Zhang, Ramsha Noor, Victoria Savalei

**Affiliations:** Department of Psychology, University of British Columbia, Vancouver, British Columbia, Canada; Goethe-Universitat Frankfurt am Main, GERMANY

## Abstract

Reverse worded (RW) items are often used to reduce or eliminate acquiescence bias, but there is a rising concern about their harmful effects on the covariance structure of the scale. Therefore, results obtained via traditional covariance analyses may be distorted. This study examined the effect of the RW items on the factor structure of the abbreviated 18-item Need for Cognition (NFC) scale using confirmatory factor analysis. We modified the scale to create three revised versions, varying from no RW items to all RW items. We also manipulated the type of the RW items (polar opposite vs. negated). To each of the four scales, we fit four previously developed models. The four models included a 1-factor model, a 2-factor model distinguishing between positively worded (PW) items and RW items, and two 2-factor models, each with one substantive factor and one method factor. Results showed that the number and type of the RW items affected the factor structure of the NFC scale. Consistent with previous research findings, for the original NFC scale, which contains both PW and RW items, the 1-factor model did not have good fit. In contrast, for the revised scales that had no RW items or all RW items, the 1-factor model had reasonably good fit. In addition, for the scale with polar opposite and negated RW items, the factor model with a method factor among the polar opposite items had considerably better fit than the 1-factor model.

## Introduction

Many Likert scales in psychology are written to contain two types of items: positively worded (PW) items and reverse worded (RW) items. PW items are phrased in the direction of the construct (e.g., the item, *I am happy*, on a scale measuring happiness), whereas RW items are phrased in the opposite direction (e.g., the item, *I am sad*, on a scale measuring happiness) [1]. RW items are used in Likert scales to reduce or eliminate acquiescence bias, which is the respondents’ tendency to agree with a given item regardless of its content [2–4]. However, in recent years, many researchers have criticized the use of RW items for several reasons (e.g., [5–7]). First, RW items can lead to confusion for respondents due to increased difficulty in interpreting the items [7–8]. Second, although RW items can control for acquiescence bias in the composite score of the scale, they cannot control for acquiescence bias in the factor structure of the scale [9]. Third, RW items may create a method factor, resulting in a scale measuring something that researchers do not intend to measure [5,10].

RW items can be categorized into two types: negated items and polar opposite items [7–8]. The former involves adding negative particles such as the word *not*; the latter involves replacing the word or phrase in the original item with that of the opposite meaning (e.g., the item, *I am sad*, on a happiness scale). The majority of RW items in psychological measures are negated items [8].

The true impact of RW items is yet to be established for a variety of psychological measures. This study focuses on the Need for Cognition (NFC) scale [11]. The NFC is theorized as a stable personality trait reflecting the person’s intrinsic motivation to engage in and enjoy cognitive activities [12]. Individuals high on the construct are likely to reflect on issues, use rational arguments, and be open to ideas [13]. The scale has been used in a variety of domains, such as health, false memory, problem solving, and advertising [12–13]. To make the scale more convenient for researchers to use, Cacioppo, Petty, and Kao [14] created an abbreviated 18-item version of the scale. This study focuses on this abbreviated 18-item NFC scale.

The 34-item NFC scale was designed to measure only one substantive construct [11]; thus, the scale should be unidimensional. However, empirical findings do not always support the purported unidimensionality of the scale. For instance, Tanaka et al., [15] identified three substantive factors in the scale: cognitive persistence, cognitive confidence and cognitive complexity; Lord and Putrevu [13] identified four factors: enjoyment of cognitive stimulation, preference for complexity, commitment of cognitive effort, and desire for understanding.

The dimensionality of the abbreviated 18-item NFC scale is also under debate. Some researchers have reasoned that the abbreviated scale should be more unidimensional than the original scale because the abbreviated scale was created by selecting the 18 items with the highest factor loadings from the original 34-item scale [14]; other researchers, however, have contended that the abbreviated scale is still multidimensional [16–18].

In addition, numerous research findings suggested that the RW items affect the factor structure of the abbreviated NFC scale. Davis et al. [17], using exploratory factor analysis, found the scale consisted of two factors: cognitive effort enjoyment and preference for problem solving. Davis et al. [17] did not provide the loadings for all items, but only listed the two items loading most highly on each factor. It is interesting to note that the two items loading most highly on the cognitive effort enjoyment factor were both RW items, and the two items loading most highly on the preference for problem solving factor were both PW items. Therefore, Davis et al. [17]’s finding may indicate that item wording is related to the structure of the scale. Bors et al. [16], Hevey et al. [18], and Forsterlee and Ho [19], using confirmatory factor analysis (CFA), found that the RW items create method effects in the abbreviated NFC scale. Forsterlee and Ho [19] and Hevey et al. [18] compared three factor models: 1) a 1-factor model without correlated errors, 2) a 1-factor model with correlated errors among the RW items, and 3) a 2-factor with one factor comprising all PW items and one factor comprising all RW items. They found that the 1-factor model without correlated errors had the worst fit, the 2-factor model had better fit, and the 1-factor model with correlated errors among the RW items had the best fit. Bors et al. [16]’s study included several other models but they also found that the model with two correlated method factors, based on item wording direction, fit much better than the model without the method factors. Finally, Furnham and Thorne [12] found that the factor structure of the scale was more unidimensional when they changed all the RW items in the scale to PW items. Similar to Davis et al. [17]’s finding, Furnham and Thorne [12], using principal component analysis, found that the original abbreviated 18-item NFC scale contained two factors distinguished by item wording directions. However, in the absence of variation in the wording direction (i.e., when all items were PW), the revised NFC scale was unidimensional.

The present study examines the abbreviated 18-item NFC scale in a similar approach to the one employed by Furnham and Thorne [12]. To build upon the limited research on the impact of RW items, our adaptation of the abbreviated NFC scale involved a total of four different scale versions: (1) the original NFC version (containing nine PW and nine RW items, of which six are polar opposite RW items and three are negated RW items), (2) the Positive version in which all items are PW, (3) the Reverse-I version in which all items are polar opposite RW items, and (4) the Reverse-II version in which all of the items are RW, but half the items are polar opposite and the other half negated. By fitting several different CFA models to each scale version, we hope to gain insight into how the scale structure changes as a result of the manipulation of the number and type of RW items.

Based on previous research (e.g., [12,16,18]), we predict that the 1-factor model will have better fit when the scale contains only PW or only RW items (Positive and Reversed-I versions). We also hypothesize that for the original scale, the 1-factor model will fit much worse than the 2-factor model that contains either two substantive factors based on wording direction or one method factor among RW or PW items. Because the Reverse-II version has never been studied, researchers know little about whether the type of the RW items affects the structure of the NFC scale. Therefore, by studying this scale version, we will explore whether the presence of different types of RW items can also cause method effects in the structure of the scale.

## Method

### Ethics Statement

Ethical approval was obtained through the University of British Columbia’s Behavioural Research Ethics Board. The written informed consent was obtained from each participant prior to the start of the study. The ethic application ID assigned by the Behavioural Research Ethics Board for this study is H13-02870.

### Participants and Procedure

A total of 1,266 undergraduate students at the University of British Columbia (1,010 female, 256 male) participated in the study for course credits. There were 312, 316, 320, and 318 respondents completing the original, Positive, Reverse-I and Reverse-II scale version, respectively. The mean age was 21 (*SD* = 3.72). On average, participants had 14.28 (*SD* = 2.00) years of education, and rated themselves 4.37 (*SD* = 0.87) on a 5-point item measuring English ability, with 1 being *poor* and 5 being *excellent*. Participants completed the study online, and then were debriefed in person. Participants were randomly assigned to complete one of the four versions of the 18-item NFC scale. In addition to the NFC scale, participants also completed a demographic questionnaire and several other psychological scales for other research studies. These psychological scales included the Beck Depression Inventory [20], the Big Five Inventory [21], the Subjective Happiness Scale [22], and the Self-Competence and Self-Liking Scale [23].

### Description of Measure

In addition to the original abbreviated scale [14], we created three revised versions, resulting in a total of four different scale versions: (1) the original NFC version, which has nine PW items, three negated RW items, and six polar opposite RW items; (2) the Positive version with all PW items; (3) the Reverse-I version with all polar opposite items; and (4) the Reverse-II version with half polar opposite and half negated items. In the revised versions, the items that did not require changing matched those in the original version (for instance, in the Positive version, the PW items from the original scale were retained and RW items were re-written to have positive wording). The revised items were created by changing the item wording direction while minimizing changes in the item content (i.e., trying to change as few words as possible). To change a PW item to a negated RW item or vice versa, *not* was either inserted or deleted. To change a PW item to a polar opposite item or vice versa, a word or phrase that has an opposite meaning was substituted. Table 1 lists all items in each scale version. The original NFC scale has 9 response options, with 1 indicating *very strong agreement* and 9 indicating *very strong disagreement*. These response options were retained for all four scale versions.

**Table 1:** Item wording for the four different versions of the abbreviated Need for Cognition (NFC) scale used in the study.

Item	Original Version	Positive Version	Reverse-I Version	Reverse-II Version
1	I would prefer complex to simple problems.	I would prefer complex to simple problems.	**I would prefer simple to complex problems.**	**I would prefer simple to complex problems.**
2	I like to have the responsibility of handling a situation that requires a lot of thinking.	I like to have the responsibility of handling a situation that requires a lot of thinking.	**I hate having the responsibility of handling a situation that requires a lot of thinking.**	***I don't like having the responsibility of handling a situation that requires a lot of thinking*.**
3	***Thinking is not my idea of fun*.**	Thinking is my idea of fun.	**Thinking is my idea of boring.**	**Thinking is my idea of boring.**
4	**I would rather do something that requires little thought than something that is sure to challenge my thinking abilities.**	I would rather do something that is sure to challenge my thinking ability than something that requires little thought.	**I would rather do something that requires little thought than something that is sure to challenge my thinking abilities.**	***I would not rather do something that is sure to challenge my thinking ability than something that requires little thought*.**
5	**I try to anticipate and avoid situations where there is likely chance that I will have to think in depth about something.**	I like to anticipate and be in situations where there is likely chance that I will have to think in depth about something.	**I try to anticipate and avoid situations where there is likely chance that I will have to think in depth about something.**	**I try to anticipate and avoid situations where there is likely chance that I will have to think in depth about something.**
6	I find satisfaction in deliberating hard and for long hours.	I find satisfaction in deliberating hard and for long hours.	**I find it frustrating to deliberate hard and for long hours.**	***I don't find satisfaction in deliberating hard and for long hours*.**
7	**I only think as hard as I have to.**	I usually think harder than I have to.	**I only think as hard as I have to.**	**I only think as hard as I have to.**
8	**I prefer to think about small, daily projects as opposed to long-term ones.**	I prefer to think about long-term projects as opposed to small, daily ones.	**I prefer to think about small daily projects as opposed to long-term ones.**	***I do not prefer to think about long-term projects as opposed to small*, *daily ones*.**
9	**I like tasks that require little thought once I’ve learned them.**	I like tasks that require a lot of thinking even after I’ve learned them.	**I like tasks that require little thought once I've learned them.**	**I like tasks that require little thought once I’ve learned them.**
10	The idea of relying on thought to make my way to the top appeals to me.	The idea of relying on thought to make my way to the top appeals to me.	**The idea of relying on thought to make my way to the top is unappealing to me.**	***The idea of relying on thought to make my way to the top does not appeal to me*.**
11	I really enjoy a task that involves coming up with new solutions to problems.	I really enjoy a task that involves coming up with new solutions to problems.	**I enjoy task that involves using already known solutions to problems.**	**I enjoy task that involves using already known solutions to problems.**
12	***Learning new ways to think does not excite me very much*.**	Learning new ways to think excites me a lot.	**Learning new ways to think makes me bored.**	***Learning new ways to think does not excite me very much*.**
13	I prefer my life to be filled with puzzles that I must solve.	I prefer my life to be filled with puzzles that I must solve.	**I prefer the puzzles in my life to have easy solutions.**	**I prefer the puzzles in my life to have easy solutions.**
14	The notion of thinking abstractly is appealing to me.	The notion of thinking abstractly is appealing to me.	**The notion of thinking abstractly is boring to me.**	***The notion of thinking abstractly is not appealing to me*.**
15	I would prefer a task that is intellectual, difficult, and important to one that is somewhat important but does not require much thought.	I would prefer a task that is intellectual, difficult, and important to one that is somewhat important but requires little thought.	**I would prefer a task that is somewhat important but requires little thought to one that is intellectual, difficult and important.**	**I would prefer a task that is somewhat important but requires little thought to one that is intellectual, difficult and important.**
16	**I feel relief rather than satisfaction after completing a task that required a lot of mental effort.**	I feel satisfaction rather than relief after completing a task that required a lot of mental effort.	**I feel relief rather than satisfaction after completing a task that required a lot of mental effort.**	***I do not feel satisfaction after completing a task that required a lot of mental effort*.**
17	***It’s enough for me that something gets the job done; I don’t care how or why it works*.**	I usually care about how or why something gets the job done.	**I usually care little about how or why something gets the job done.**	**I usually care little about how or why something gets the job done.**
18	I usually end up deliberating about issues even when they do not affect me personally.	I usually end up deliberating about issues even when they do not affect me personally.	**I usually only deliberate about issues that affect me personally.**	***I usually don't end up deliberating about issues when they do not affect me personally*.**

*Note*. RW items are bolded. Of the RW items, the italicized are negated; the rest are polar opposite. The original NFC scale (Cacioppo, Petty & Kao, 1984) contains nine PW items, which are items 1, 2, 6, 10, 11, 13, 14, 15, and 18, and nine RW items, which are items 3, 4, 5, 7, 8, 9, 12, 16, and 17. The Positive-I Version contains all PW items. The Reverse-I Version contains all polar opposite RW items. The Reverse-II Version contains nine polar opposite items, which are items 1, 3, 5, 7, 9, 11, 13, 15, and 17, and nine negated items, which are items 2, 4, 6, 8, 10, 12, 14, 16, and 18.

### Data Analysis

Using the *psych* package (Revelle, 2014) [24] in R, we conducted parallel analyses to compare the dimensionalities of the four versions of the NFC scale. Parallel analysis is better than the traditional scree plot analysis because it incorporates sampling variability into the scree plot [25]. In parallel analysis, the scree plot obtained from the data is compared to an average scree plot obtained from a simulated dataset generated from a population where all variables are uncorrelated [26–27]. The number of factors is estimated by counting the number of eigenvalues in the data that are greater than the corresponding eigenvalues obtained from the simulated dataset.

In addition, we conducted exploratory factor analyses (EFAs) of the four scale versions. To examine whether the item wording direction or item content affected the scale’s factor structure, we extracted two factors for each scale version. The EFA extraction method was least squares (a.k.a minres), followed by an oblimin rotation. Finally, we conducted confirmatory factor analyses (CFAs) for each scale version using the *lavaan* package in *R* [28]. We used the ML estimator with Satorra-Bentler corrections for nonnormality (i.e., estimator = “mlm”) because Mardia’s kurtosis tests indicated that the data for all four scale versions were significantly nonnormal [29–30].

For each scale version, we fit four different models (see factor loadings tables in the Results section for the items that load on each factor in each scale version). Model 1 is a 1-factor model with all items loading on one substantive factor. Model 2 is a 2-factor model with two correlated substantive factors. For the original, Positive and Reverse-I versions, the two factors in Model 2 were formed based on whether the item is PW or RW in the original version (i.e., item wording direction). For the Reverse-II version, the two factors in Model 2 were formed based on whether the item is polar opposite or negated (i.e., RW item type). Models 3 and 4 are both 2-factor models, each model having one substantive factor comprising all items and one method factor. In both models, the correlation between the substantive factor and the method factor was set to zero. For the original, Positive and Reverse-I versions, the RW items from the original version loaded on the method factor in Model 3, and the PW items from the original version loaded on the method factor in Model 4. For the Reverse-II version, the polar opposite and negated items loaded on the method factor in Models 3 and 4, respectively. Model 1 is the correct theoretical model for the NFC scale [11]. Model 2 has been examined in various research studies on the factor structure of the NFC scale [12,16,18,19]. Models 3 and 4 have been previously used to study method effects in other Likert scales that are not NFC measures [5,10]. We did not explore models with two method effects (i.e., where each item is also an indicator of a method factor) because these models often suffer from identification problems [31,32]. Additionally, researchers who consider both one and two method effects for RW/PW items in other psychological scales tend to find that these models fit similarly (e.g., [5,33]).

Three fit indices were examined for each model: robust chi-square (*χ*^2^; [29]), Comparative Fit Index (CFI) with a nonnormality correction [34,35], and Root Mean Square Error of Approximation (RMSEA) with a nonnormality correction [36,37]. A reasonably well-fitting model should have a CFI value of 0.90 or greater [38] and an RMSEA value of 0.08 or lower [36].

## Results

Item means and standard deviations from all NFC scale versions are presented in Table 2. In general, the item means and standard deviations across the four versions of the NFC scale were similar (see Table 2). The original version had slightly lower internal consistency than the other three scales: Cronbach’s alpha values were 0.88, 0.95, 0.94 and 0.91 for the original, Positive, Reverse-I, and Reverse-II versions respectively.

**Table 2:** Descriptive Statistics for all versions of Need for Cognition (NFC) scale.

	Original Version		Positive Version		Reverse-I Version		Reverse-II Version	
	*M*	*SD*	*M*	*SD*	*M*	*SD*	*M*	*SD*
Item 1	4.65	2.08	4.66	2.02	5.73	2.03	5.69	2.01
Item 2	4.12	1.88	4.04	1.85	4.78	2.09	4.49	1.95
Item 3	4.52	1.99	4.23	1.94	3.03	1.77	2.84	1.75
Item 4	4.69	1.97	4.02	1.83	4.01	1.98	4.54	2.20
Item 5	4.15	1.89	4.08	1.85	3.77	1.93	3.80	2.01
Item 6	4.75	1.95	4.74	1.97	4.51	2.10	4.28	2.05
Item 7	5.40	2.07	3.63	1.76	4.80	2.11	4.91	2.21
Item 8	5.43	1.95	4.30	1.90	5.18	2.07	4.52	2.06
Item 9	5.68	1.80	4.57	1.79	5.23	2.08	5.28	2.00
Item 10	3.78	1.57	4.00	1.76	3.99	1.97	3.81	1.86
Item 11	3.78	1.60	3.94	1.79	5.37	2.04	5.43	1.90
Item 12	4.08	1.93	3.75	1.91	3.38	1.89	3.41	1.81
Item 13	4.73	1.80	4.50	1.81	5.35	2.01	5.39	1.89
Item 14	4.31	1.81	4.13	1.91	3.68	1.92	4.22	1.97
Item 15	4.14	1.73	4.12	1.80	4.51	2.09	4.56	1.93
Item 16	5.36	2.05	3.58	1.93	4.52	2.25	2.53	1.75
Item 17	4.52	2.06	3.53	1.71	3.81	2.00	3.34	1.84
Item 18	3.77	1.75	3.62	1.72	4.51	2.14	4.11	2.07
Average	4.55	1.88	4.08	1.85	4.45	2.03	4.29	1.96

### Parallel Analysis

**Fig 1:**
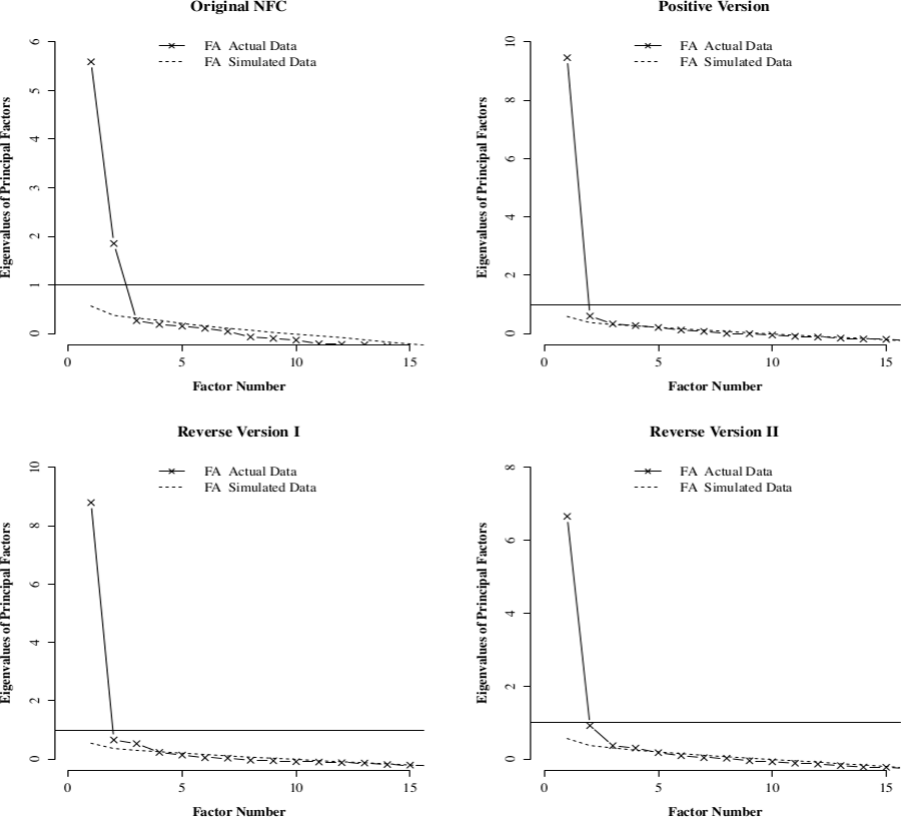
Parallel analyses for the four version of the NFC scale. In each graph, the horizontal line indicates the eigenvalue of one. The straight line is the scree plot obtained from the data. The dashed line is an average scree plot obtained from simulated dataset with the same dimension as the original dataset but generated from a population where all variables are uncorrelated.

Fig 1 shows the results from the parallel analyses of the four NFC scale versions. Overall, as expected, the parallel analyses suggested that the original version had higher dimensionality than the other three scale versions. Specifically, for the original version, the parallel analysis plot clearly indicated two factors. For the Positive and Reverse-I versions, although more than one eigenvalues were above the scree plot obtained from the simulated data, only one eigenvalue in each of the two plots was well above the scree plot; the other eigenvalues were very close to the scree plot. For the Reverse-II version, the parallel analysis plot indicated two factors but the second eigenvalue in the Reverse-II version was much smaller than the second eigenvalue in the original version.

### Exploratory Factor Analyses

The estimated loadings and factor correlations for the four scale versions are shown in Table 3. The original scale had the cleanest two-factor solution: each item loaded high on one factor and low on the other factor, and the factor correlation between the two factors was relatively low. In contrast, in the other three versions, some items loaded equally high on both factors, and the factor correlations between the two factors were relatively high, suggesting that the two factors were not as distinct.

**Table 3:** Standardized Factor Loadings of Model 2 for the Four Scale Versions.

	Original version		Positive Version		Reverse-I Version		Reverse-II Version	
	Factor 1	Factor 2	Factor 1	Factor 2	Factor 1	Factor 2	Factor 1	Factor 2
Item 1	-0.01	**0.76**	**0.80**	-0.18	0.12	**0.59**	**0.51**	0.12
Item 2	0.08	**0.73**	**0.85**	-0.04	-0.11	**0.89**	***0*.*40***	*0*.*37*
Item 3	**0.65**	0.17	**0.86**	-0.06	0.07	**0.69**	0.09	**0.68**
Item 4	**0.69**	0.24	**0.89**	-0.07	0.15	**0.73**	-0.12	**0.64**
Item 5	**0.72**	0.08	**0.83**	-0.01	0.03	**0.77**	0.25	**0.57**
Item 6	0.12	**0.53**	**0.71**	0.09	0.13	**0.64**	***0*.*40***	*0*.*34*
Item 7	**0.71**	-0.14	0.07	**0.56**	**0.63**	0.12	**0.68**	0.03
Item 8	**0.67**	-0.28	0.00	**0.71**	**0.71**	-0.06	***0*.*40***	*0*.*27*
Item 9	**0.53**	0.18	**0.52**	0.41	**0.82**	-0.01	**0.82**	-0.10
Item 10	0.09	**0.64**	**0.64**	0.21	***0*.*47***	*0*.*34*	0.27	**0.50**
Item 11	0.06	**0.66**	**0.76**	0.11	**0.81**	-0.11	**0.77**	-0.09
Item 12	**0.62**	0.07	**0.62**	0.26	***0*.*44***	*0*.*33*	0.12	**0.66**
Item 13	-0.07	**0.73**	**0.80**	-0.07	**0.72**	-0.08	**0.58**	0.01
Item 14	0.04	**0.64**	**0.69**	0.11	*0*.*38*	***0*.*40***	*0*.*41*	***0*.*44***
Item 15	-0.01	**0.70**	**0.76**	0.07	**0.66**	0.23	**0.54**	0.34
Item 16	**0.53**	0.01	**0.56**	0.19	**0.53**	0.18	-0.23	**0.78**
Item 17	**0.74**	-0.02	*0*.*38*	***0*.*42***	**0.47**	0.29	-0.02	**0.63**
Item 18	-0.08	**0.41**	*0*.*33*	***0*.*43***	**0.74**	-0.02	***0*.*35***	*0*.*33*
Factor Correlation	0.38		0.52		0.72		0.52	

*Note*: For each item in each version, the highest loading was bolded, and loadings that are relatively equally high on both factors are italicized.

For the original version, the two extracted factors corresponded to the item wording direction: all RW items loaded on Factor 1, and all PW items loaded on Factor 2. For the Reverse-II version, the two factors were related to the type of RW items. The polar opposite RW items tended to load on Factor 1, and the negated RW items tended to load on Factor 2. For the Positive and Reverse-I version, the two factors were mainly formed based on the item content. For the Positive version, only two items (Items 7 and 8) loaded high on Factor 2; therefore, Factor 2 did not seem to be a meaningful factor. Factor 2 may have emerged due to the similar wording of Items 7 and 8 (i.e., they both contained the word *think*). For the Reverse-I version, the high factor correlation made it difficult to interpret the two factors; however, items that tended to load on the same factor also shared similar wording. For example, Items 3, 12 and 14, which loaded relatively high on Factor 2, all contained the words *bored* or *boring*.

### Confirmatory Factor Analysis

The standardized factor loadings of each model for each scale version are presented in Tables 4–7. Robust chi-square difference tests between Model 1 and each of the Models 2, 3, and 4 were also conducted (Models 1 is nested within the other three models); however, because chi-square difference tests are not appropriate when the less restricted baseline model (i.e., Models 2, 3, or 4) does not fit the data [39], we do not present the results from the chi-square difference tests in the main manuscript. Readers who are interested in these results can refer to Table in S1 Table. Several patterns in the factor loadings suggested that the original version was less unidimensional compared to the other versions. First, even though all the loadings for Model 1 were significant at α = 0.05 for all scale versions, the loading sizes for the original version were somewhat lower than those for the other versions. The average loading size of Model 1 for the original version was 0.55, but the average loadings for the Positive, Reverse-I and Reverse-II versions ranged from 0.60 to 0.72. Second, the factor correlation in Model 2 was lower for the original version than for the revised versions. For the original version, the factor correlation in Model 2 was 0.56; however, for the Positive, Reverse-I and Reverse-II versions, the factor correlations ranged from 0.96 to 0.99. Finally, for the original version, the loadings on the method factors in Models 3 and 4 were large and all positive; however, for the other versions, the loadings were generally small and sometimes negative. In particular, for the original version, the average value of all the loadings on the method factors in Models 3 and 4 was 0.54. In contrast, for the Positive, Reverse-I and Reverse-II versions, the average loading value on the method factors was 0.20; and six, nine and two of the 18 loadings were negative for the three scales, respectively.

**Table 4:** Standardized Factor Loadings of Model 1 for the Four Scale Versions.

	Original version	Positive Version	Reverse-I Version	Reverse-II Version
	NFC Factor	NFC Factor	NFC Factor	NFC Factor
Item 1	0.60	0.66	0.62	0.52
Item 2	0.66	0.81	0.69	0.66
Item 3	0.68	0.82	0.70	0.68
Item 4	0.77	0.84	0.81	0.40
Item 5	0.67	0.82	0.73	0.70
Item 6	0.51	0.75	0.69	0.61
Item 7	0.44	0.41	0.69	0.57
Item 8	0.29	0.47	0.60	0.55
Item 9	0.57	0.78	0.75	0.60
Item 10	0.62	0.79	0.76	0.67
Item 11	0.59	0.83	0.64	0.58
Item 12	0.56	0.78	0.72	0.69
Item 13	0.54	0.74	0.59	0.50
Item 14	0.55	0.76	0.72	0.76
Item 15	0.55	0.80	0.84	0.77
Item 16	0.43	0.66	0.63	0.47
Item 17	0.57	0.64	0.70	0.52
Item 18	0.25	0.58	0.66	0.55
Average	0.55	0.72	0.70	0.60

**Table 5:** Standardized Factor Loadings of Model 2 for the Four Scale Versions.

	Original version		Positive Version		Reverse-I Version		Reverse-II Version	
	PW Item Factor	RW Item Factor	PW Item Factor	RW Item Factor	PW Item Factor	RW Item Factor	Polar Opposite Item Factor	Negated Item Factor
Item 1	0.74		0.66		0.62		0.53	
Item 2	0.76		0.81		0.69			0.66
Item 3		0.75		0.82		0.69	0.68	
Item 4		0.84		0.84		0.81		0.40
Item 5		0.79		0.83		0.72	0.70	
Item 6	0.54		0.75		0.68			0.61
Item 7		0.55		0.41		0.69	0.58	
Item 8		0.43		0.47		0.60		0.56
Item 9		0.58		0.79		0.75	0.61	
Item 10	0.69		0.79		0.76			0.68
Item 11	0.69		0.83		0.64		0.59	
Item 12		0.61		0.78		0.72		0.70
Item 13	0.66		0.75		0.58		0.51	
Item 14	0.63		0.76		0.72			0.76
Item 15	0.67		0.80		0.83		0.78	
Item 16		0.48		0.66		0.63		0.48
Item 17		0.65		0.65		0.70	0.52	
Item 18	0.33		0.58		0.66			0.52
Average	0.63	0.63	0.75	0.69	0.69	0.70	0.61	0.60
Factor Correlation	0.56		0.98		0.99		0.96	

**Table 6:** Standardized Factor Loadings of Model 3 for the Four Scale Versions.

	Original version		Positive Version		Reverse-I Version		Reverse-II Version	
	NFC Factor	RW Method Factor	NFC Factor	RW Method Factor	NFC Factor	RW Method Factor	NFC Factor	Polar Opposite Method Factor
Item 1	0.74		0.65		0.63		0.49	0.22
Item 2	0.76		0.81		0.68		0.65	
Item 3	0.45	0.59	0.82	*-0*.*12*	0.69	0.21	0.73	-0.20
Item 4	0.54	0.63	0.85	*-0*.*12*	0.81	0.38	0.41	
Item 5	0.41	0.67	0.83	*-0*.*16*	0.72	0.34	0.71	*0*.*02*
Item 6	0.54		0.76		0.69		0.59	
Item 7	0.18	0.57	0.40	0.31	0.70	-0.17	0.54	0.26
Item 8	*0*.*03*	0.53	0.46	0.50	0.61	-0.21	0.54	
Item 9	0.41	0.43	0.79	0.35	0.76	-0.16	0.54	0.53
Item 10	0.69		0.78		0.76		0.68	
Item 11	0.69		0.82		0.65		0.51	0.62
Item 12	0.35	0.51	0.77	*0*.*08*	0.72	*-0*.*02*	0.71	
Item 13	0.67		0.74		0.59		0.45	0.34
Item 14	0.63		0.75		0.72		0.76	
Item 15	0.67		0.80		0.84		0.74	0.21
Item 16	0.25	0.41	0.65	*0*.*07*	0.63	*-0*.*07*	0.51	
Item 17	0.32	0.58	0.63	*0*.*21*	0.70	*-0*.*10*	0.55	-0.15
Item 18	0.33		0.57		0.66		0.55	
Average	0.48	0.55	0.72	0.21	0.70	0.18	0.59	0.28

*Note*. Italicized loadings are not significant at α = 0.05. The average loading values were calculated based on absolute values of the loadings. For each scale, the correlation between the NFC factor and the method factor was fixed to zero.

**Table 7:** Standardized Factor Loadings of Model 4 for the Four Scale Versions.

	Original version		Positive Version		Reverse-I Version		Reverse-II Version	
	NFC Factor	PW Method Factor	NFC Factor	PW Method Factor	NFC Factor	PW Method Factor	NFC Factor	Negated Method Factor
Item 1	0.38	0.64	0.64	0.73	0.63	-0.24	0.53	
Item 2	0.46	0.61	0.80	0.24	0.71	-0.48	0.66	*0*.*01*
Item 3	0.75		0.82		0.70		0.67	
Item 4	0.84		0.84		0.81		0.38	0.18
Item 5	0.79		0.82		0.73		0.70	
Item 6	0.37	0.40	0.75	*0*.*04*	0.69	-0.18	0.60	*0*.*07*
Item 7	0.55		0.41		0.68		0.59	
Item 8	0.43		0.47		0.59		0.54	*0*.*07*
Item 9	0.58		0.79		0.75		0.62	
Item 10	0.44	0.52	0.80	-0.15	0.76	*0*.*12*	0.65	0.20
Item 11	0.40	0.56	0.83	*0*.*02*	0.64	0.14	0.60	
Item 12	0.61		0.78		0.72		0.66	0.31
Item 13	0.31	0.60	0.73	0.15	0.59	0.18	0.51	
Item 14	0.37	0.51	0.76	*-0*.*01*	0.72	*0*.*12*	0.76	*0*.*03*
Item 15	0.33	0.60	0.80	*0*.*00*	0.84	0.17	0.78	
Item 16	0.48		0.66		0.63		0.41	0.71
Item 17	0.65		0.64		0.70		0.50	
Item 18	*0*.*12*	0.33	0.58	*-0*.*06*	0.66	0.18	0.55	*0*.*07*
Average	0.49	0.55	0.72	0.15	0.70	0.20	0.59	0.18

*Note*. Italicized loadings are not significant at α = 0.05. The average loading values were calculated based on absolute values of the loadings. For each scale, the correlation between the NFC factor and the method factor was fixed to zero.

The fit statistics for the four models for all four scale versions are presented in Table 8. Consistent with previous findings [12,16,18–19], for the original NFC scale, the 1-factor Model 1 had poor fit; however, Models 2–4, which contained either two substantive factors based on wording direction or one method factor for RW or PW items, had reasonably good fit. Specifically, when Model 1 was fit to data from the original NFC scale, the robust CFI and RMSEA values were 0.71 and 0.11, respectively; however, when Models 2, 3 and 4 were fit to the same data, the robust CFI and RMSEA values were around 0.90 and 0.06, respectively. Even though all four models failed to fit the data by the chi-square test, the values of the Satorra-Bentler chi-square statistic were much lower for Models 2–4 than for Model 1.

**Table 8:** CFA results for the four models fit to the four scale versions.

	*χ*^2^	*df*	CFI	RMSEA
** **	**Original Version**			
Model 1	611.83	135.00	0.70	0.12
Model 2	296.64	134.00	0.91	0.07
Model 3	262.27	126.00	0.92	0.06
Model 4	283.87	126.00	0.91	0.07
	**Positive Version**			
Model 1	418.36	135.00	0.89	0.10
Model 2	417.95	134.00	0.90	0.10
Model 3	370.56	126.00	0.91	0.09
Model 4	366.42	126.00	0.91	0.09
	**Reverse-I Version**			
Model 1	397.42	135.00	0.89	0.09
Model 2	395.56	134.00	0.89	0.09
Model 3	353.90	126.00	0.91	0.09
Model 4	363.04	126.00	0.90	0.09
	**Reverse-II Version**			
Model 1	344.77	135.00	0.86	0.09
Model 2	343.89	134.00	0.86	0.09
Model 3	243.66	126.00	0.92	0.07
Model 4	319.25	126.00	0.87	0.08

*Note*: The *χ*^2^ values were corrected for nonnormality using the Satorra-Bentler’s correction [29]. The CFI and RMSEA values were also corrected for nonnormality using equations in Brosseau-Liard and Savalei [35], and Brosseau-Liard, Savalei and Li [37].

The Positive and Reverse-I scale versions seem to be more unidimensional than the original scale version. Model 1 had much better fit for the Positive and Reverse-I scale versions than for the original scale version, although the robust CFI and RMSEA values were slightly below the acceptable cutoff points. For the Positive and Reverse-I versions, the fit of Models 2–4 was slightly better than the fit of Model 1, but this is to be expected because Models 2–4 contained fewer fixed parameters than Model 1.

For the Reverse-II scale version, Models 1, 2, and 4 had similar fit but Model 3, which had a method factor for the polar opposite items, had better fit than the other models. For Models 1, 2 and 4, the robust CFI and RMSEA values were in the 0.86–0.87 and 0.08–0.09 ranges, respectively, but for Model 3, the robust CFI and RMSEA values were 0.92 and 0.07, respectively. Although Model 3 had good fit, most loadings on Model 3’s method factor for the polar opposite items were not large (see Table 6). Of the nine loadings on the method factor in Model 3, six loadings had absolute values less than 0.30; and two of these six loadings were negative. These results suggested that the factor structure of Reverse-II version may be affected by a method effect due to the polar opposite items but this method effect may not be too strong.

## Discussion

The primary purpose of this study was to examine how the number and type of RW items affect the factor structure of the abbreviated 18-item NFC scale. Consistent with our hypotheses, for the original scale with both PW and RW items, the fit of the 1-factor model (i.e., Model 1) was poor, but the fit of the 2-factor models (i.e., Models 2–4) was reasonably good. In contrast, in the Positive and Reverse-I versions, the fit of the 1-factor model was reasonably good, and the fit of the 2-factor models was similar to that of the 1-factor model. Furthermore, the loadings on the NFC factor in the 1-factor model were lower for the original scale version than for the other versions, but the loadings on the method factor in the 2-factor models (i.e., Models 3 and 4) were much higher for the original version than for the other versions. Therefore, building on previous research, our findings lend further support to the idea that the RW items cause method effects and multidimensionality in the abbreviated NFC scale [12,16,18].

Our study has also found that when the scale contained different types of RW items (i.e., in the Reverse-II version), adding a method factor among the polar opposite items improved model fit considerably, relative to both the 1-factor model (Model 1) and the model with a method factor for negated RW items (Model 3). This finding suggests that the type of RW items may also affect the factor structure of the NFC scale. One possible explanation is that the cognitive processes for making inconsistent responses (i.e., agreeing with both PW and RW items) in the presence of polar opposite RW items are different from those in the presence of negated RW items. According to Weijters and Baumgartner [40], the main reason for inconsistent response in the presence of polar opposite items is that participants may not interpret the antonyms (or other phrases) used in the RW items as contradictory to the construct of interest, and thus may agree with both PW and RW items. For example, a participant may agree with both the items *I like simple tasks* and *I like complex tasks* because *liking simple tasks* does not necessarily imply *not liking complex tasks*. This problem of polar opposite RW item is called reversal ambiguity. Negated RW items do not have the problem of reversal ambiguity but they may cause careless responding or judgmental difficulty for some participants. In the presence of negated items, participants may miss the presence of a negative particle in the item (e.g., misread *I am not happy* as *I am happy*), making errors due to carelessness [7]. Participants may also find it difficult to assess their level of agreement when the item contains a negation [8,40]. In other words, a negated item makes it more difficult for the participant to judge whether the item content is consistent with the participant’s own beliefs (see [8] for a detailed explanation). When a scale contains both polar opposite and negated RW items, some participants may make inconsistent response due to the reversal ambiguity of the polar opposite items whereas others may make inconsistent response due to the judgmental difficulty of or careless responding to the negated items; as a consequence, method effects due to the types of RW items will emerge.

Although the factor structure of the Reverse-II scale seems to be affected by a method factor for the polar opposite items, the loadings on this method factor were generally small, suggesting the method effect due to the type of RW item may not be too strong. Further studies are needed to fully understand the similarities and differences between polar opposite and negated RW items. Future research should examine how different types of RW items affect the factor structure of other psychological scales. Researchers can also use qualitative methods (e.g., the think-aloud approach by Lewis [41]) to examine respondents’ cognitive processes when responding to different types of RW items.

The fact that the factor structure of the NFC scale changes with the number and type of RW items may have important implications for the use of this scale. Further, as there is no reason to assume that the NFC scale is affected by RW items any more so than any other scale, this is a larger problem with all Likert scales containing PW and RW items. For instance, Van Sonderen et al.[7] showed that correlations between pairs of items are often higher when they are worded in the same direction than when they measure the same construct. Thus, correlations between the NFC scale and other psychological scales may be affected by the number and type of RW items on both, compromising validity assessments via convergence and discriminant validity estimates. Further research should investigate the extent to which item wording influences validity estimates obtained by correlating different versions of the NFC scale with other important scales in its nomological network.

In summary, the use of RW items in Likert scales, while extremely common, has serious disadvantages. RW items can contaminate the factor structure of the scale so that more complex models (introducing method factors) will be necessary to achieve good fit. Researchers unfamiliar with such models may reach the false conclusion that the substantive factor of interest is multidimensional [32]. While the present study does not establish conclusively that the abbreviated 18-item NFC scale is unidimensional, it does show that the approximation of the scale’s structure by the 1-factor model is much better for the three alternate versions of the scale than for the original version. Our study also shows that the factor structures of Likert scales are very susceptible to the presence of different item types—even when all items are RW. Alternative item formats, such as the recently proposed Expanded scale format [42], do a much better job controlling acquiescence bias while removing the sensitivity to item type. We hope that our study can contribute to making researchers aware of the drawbacks associated with RW items. We also encourage further studies of the effect of RW items on the structure of different psychological scales, resulting in more effective and accurate measurement across different domains in social sciences.

## Supporting Information

S1 Table Robust Chi-Square Difference Tests. The robust chi-square difference tests are conducted according to Satorra and Bentler’s approach. ns = not significant (i.e., *p*-value greater than 0.05); *** = *p*-value less than 0.001.
(DOCX)
