# Supplementary material for: Examining the Effect of Reverse Worded Items on the Factor Structure of the Need for Cognition Scale
Source: PLoS One. 2016 Jun 15;11(6):e0157795. doi: 10.1371/journal.pone.0157795 (PMC4909292; doi:10.1371/journal.pone.0157795)
Supplement: S1 Table — The robust chi-square difference tests are conducted according to Satorra and Bentler’s approach. ns = not significant (i.e., p-value greater than 0.05); *** = p-value less than 0.001. (DOCX) [file pone.0157795.s001.docx]

**S1 Table: Robust Chi-Square Difference Tests:**

|  | Original | Positive | Reverse I | Reverse II |
| --- | --- | --- | --- | --- |
| Δ*χ^2^* _model1-model2_ (*df*=1) | 25.81*** | 1.76 *ns* | 2.17 *ns* | 1.33 *ns* |
| Δ*χ^2^* _model1-model3_ (*df*=9) | 154.59*** | 39.18*** | 37.62*** | 86.84*** |
| Δ*χ^2^* _model1-model4_ (*df*=9) | 164.05*** | 51.98*** | 31.81*** | 25.25*** |

*Note:* The robust chi-square difference tests are conducted according to Satorra and Bentler [1]’s approach. ns= not significant (i.e., *p*-value greater than 0.05); *** = *p*-value less than 0.001

**Reference:**

1. Satorra A, Bentler PM. A scaled difference chi-square test for moment structure analysis. Psychometrika. 2001;66;507-14. doi: 10.1007/BF02296192
